# Supplementary material for: 2D Collagen Membranes from Marine Demosponge Chondrosia reniformis (Nardo, 1847) for Skin-Regenerative Medicine Applications: An In Vitro Evaluation
Source: Mar Drugs. 2023 Jul 28;21(8):428. doi: 10.3390/md21080428 (PMC10455478; doi:10.3390/md21080428)
Supplement: Supplementary file 1 [file marinedrugs-21-00428-s001.zip › marinedrugs-2463067-supplementary.pdf]

Table S1. Primer sequences used in the qPCR analyses.

Gene	GenBank ID	Forward	Reverse
**GAPDH**	M32599	5′-TCTCCCTCACAATTTCCATCCCAg-3′	5′-gggTgCAgCgAACTTTATTgATgg-3′
**COL1A1**	NM_007742.4	5′-CTgCTggTCCTgCTggTC-3′	5′-CCTTgTTCgCCTGTCTCAC-3′
**FN**	NM_001276412	5′-CCAgTTCAgAggAgCATCAg-3′	5′-ggCATTgTCgTTCAgAgTgTA-3′
**MMP3**	NM_010809.3	5′-TgACgATgATgAACgATggA-3′	5′-CCTTggCTgAgTggTAgAg-3′
**TGF-β**	NM_011577.2	5′-AATTCCTggCgTTACCTT-3′	5′-CCTgTATTCCgTCTCCTT-3′
**FGF2**	NM_008006.2	5′-CTACAACTCCAAgCAgAAgA-3′	5′-gTTATTAgATTCCAgTCgTTCAA-3′
**IL-1β**	NM_008361	5′-AgTgATgAgAATgACCTgTT-3′	5′-gATACTgCCTgCCTgAAg-3′
